# Investigation into the Effect of Varied Functional Biointerfaces on Silicon Nanowire MOSFETs

**DOI:** 10.3390/s121216867

**Published:** 2012-12-06

**Authors:** Shu-Ping Lin, Tien-Yin Chi, Tung-Yen Lai, Mao-Chen Liu

**Affiliations:** 1 Graduate Institute of Biomedical Engineering, National Chung Hsing University, 250 Kuo-Kuang Road, Taichung 40227, Taiwan; E-Mail: angeldtf@hotmail.com; 2 National Nano Device Laboratories, 26 Prosperity Road 1, Hsinchu Science Park, Hsinchu 30078, Taiwan; E-Mails: dylai@ndl.narl.org.tw (T.-Y.L.); iwfn6727@mail2000.com.tw (M.-C.L.)

**Keywords:** functional biointerface, surface modification, electrical measurement, pH sensing, protein interaction

## Abstract

A biocompatible and functional interface can improve the sensitivity of bioelectronics. Here, 3-aminopropyl trimethoxysilane (APTMS) and 3-mercaptopropyl trimethoxysilane (MPTMS) self-assembled monolayers (SAMs) were independently modified on the surface of silicon nanowire metal-oxide-semiconductor field effect transistors (NW-MOSFETs). Those SAMs-modified silicon NW-MOSFETs were used to discriminate various pH solutions and further verify which modified regime was capable of providing better electrical signals. The APTMS-SAM modified NW-MOSFETs showed better electrical responses in pH sensing. Biomolecules on APTMS-SAM modified NW-MOSFETs also gave better signals for the corresponding proteind in physiological buffer solutions. Atomic force microscopy (AFM) clarified those electrical phenomena and found biomolecules on APTMS-SAM were relatively uniformly modified on NW-MOSFETs. Our results showed that more uniform modification contributed to better signal response to protein interactions in physiological buffer solutions. It suggests that suitable surface modifications could profoundly affect the sensing response and sensitivity.

## 1. Introduction

Metal-oxide-semiconductor field-effect transistors (MOSFETs) have been developed by using advanced micro- and nano-electro-mechanical systems (M/NEMS) technology [1–3]. Recently, various nanowire-based field-effect transistors (NW-FETs) have been explored in many biomedical aspects because of their high selectivity, extreme sensitivity, rapid response, and potential for integration into full electronic on-chip systems for high-throughput biological analysis [2,4–6]. The sensing response and sensitivity of NW-FETs are sensitive to several factors, such as an appropriate technique to modify and functionalize the surface, Debye length, and the presence of efficient amounts of the corresponding molecules, *etc.* However, there is a lack of studies investigating those factors other than Debye length. It is difficult to sense molecules in physiological buffer solution with NW-based sensors due to the shorter Debye length. There have been lots of investigations on changing the strength of solvents to increase the Debye length and procure a better signal [7,8]. So far, many label-free sensing NW devices have utilized controlled buffers to perform measurements, for example, low salt buffers were required to prevent screening of the charge-based electronic signal [2,4–8]. Although controlled buffers have addressed the deterioration of the detection sensitivity of NW-FET, biomolecules might denature in such severe environments. The development of a hybrid nanoelectronic enzyme-linked immunosorbent assay (ne-ELISA) that combines the enzymatic conversion of a bound substrate with NW-FETs demonstrated a satisfying sensing sensitivity in physiological buffer solution without affecting the inherent properties of proteins [9].

Since biofouling and non-specific binding significantly influence the minute active sites on the surface of NWs under physiological buffer solution conditions [8], an appropriate and effective functionalization of the sensing area needs to be further investigated [10,11]. Surface modification techniques have been applied to functionalize FETs for the specific detection of ions [4,12], DNA [7,13], proteins [4,8], and viruses [14]. In addition, the surface modification or functionalization introduces functional groups and then creates an interface between the biological system and the electronic device for detection of neuronal signals [15] and observation of cell growth [16]. Organosilane molecules are popular candidates for reacting with hydroxyl-terminated surfaces [17]. When cleaned silicon substrates are exposed to an ambient environment, silicon oxide layer spontaneously grow on the outermost substrates [17]. Silanization is the most applied method of modifying silicon oxide by creating a uniform self-assembled monolayer (SAM) of silanol groups, which can further conjugate biomolecules to form a functional biointerface. This biointerface with tunable biocompatibility can contribute better electrical signals to specific detection problems. There have been lots of studies that have exploited the functional biointerfaces on silicon NW-FET for many biological tests, such as protein interactions [2,4,8] and specific antigen-antibody reactions [5,6,18]. One of the most important features of proteins is their ability to interact specifically with other ones, and such protein-protein interactions play pivotal roles in a variety of cellular functions and physiological conditions. Therefore, significant progress in developing cancer-screening sensors by observing this interaction *in vitro* has been implemented by using silicon NW-FETs [2,5,6,9,19].

Although various studies have tackled surface modification of silicon NW, the comparison of the effect of variously modified SAM molecules on sensing signals is seldom addressed. In this study, 3-aminopropyl trimethoxysilane (APTMS) and 3-mercaptopropyl trimethoxysilane (MPTMS) SAMs were independently created on the silicon oxide surface of nanowire MOSFETs (NW-MOSFETs). The amino (-NH_2_) groups of APTMS and mercapto (-SH) groups of MPTMS contributed a functional interface for pH sensing. In order to verify which SAM molecule has a greater impact on the sensitivity of silicon NW-MOSFETs, the investigation of antibody-antigen reaction was performed after biomolecules were subsequently immobilized on each SAM. The appearance of each step of the modification and immobilization process was scanned by AFM and matched to the results of electrical measurement in pH tests and detection of various concentrations of biomolecules.

## 2. Experimental Section

### 2.1. Fabrication of NW-MOSFETs

**Figure 1.**
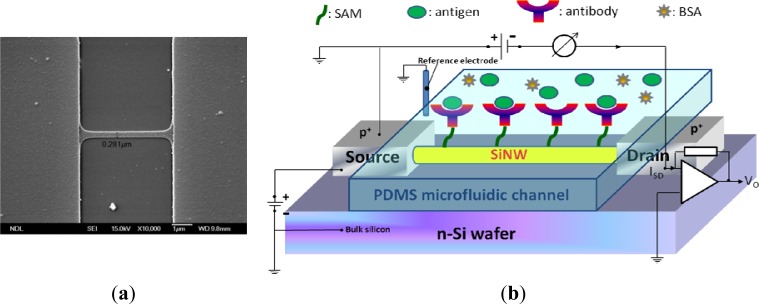
(**a**) The top-view SEM image of the NW-MOSFETs with a NW width of approximately 280 nm. (**b**) The setup of electrical measurement system in our study.

Poly-Si MOSFET NWs were manufactured on standard 6-in. phosphorus-doped n-type wafers. A 100-nm polysilicon layer was deposited using the CVD process. Subsequently, the poly-Si wire was patterned by the standard I-line stepper of the MOS semiconducting process. The photoresist trimming and polysilicon etch were respectively performed in a transformer coupled plasma (LAM TCP 9400 SE) with classical HBr/Cl_2_/O_2_ chemistry, the width of NWs was scaled to a level of approximate 280 nm. A channel protection photoresist pattern was then formed by I-line lithography to keep the channel intrinsically from source/drain (S/D) implantation. Subsequently, the S/D pads were doped with a 1 × 10^22^ cm^−3^ boron ion at 35 keV to reduce the parasitic resistance of the NW. Thereafter, the photoresist on channel protection was removed. The S/D dopants were activated by a rapid thermal anneal step at 900 °C for 30 min in N_2_ ambiance. A top-view SEM image of the NW is shown in Figure 1(a). Metal contacts were created by deposition of Ni/Ti layers on S/D pads. The devices were then annealed at 450 °C for 30 min in N_2_ ambiance to construct a reliable metal/silicon ohmic contact. Polydimethylsiloxane (PDMS) microfluidic channels (15 mm × 0.7 mm × 0.2 mm) were fabricated using standard MEMS lithography technology and used to introduce all the solutions in this study.

### 2.2. Surface Modification

First, NW-MOSFETs were independently washed with 10% HCl solution and 10% NaOH solution. After that, NW-MOSFETs were then rinsed by pure ethanol. The NWs were respectively modified with 1% ethanolic solution of APTMS (H_2_N(CH_2_)_3_Si(OCH_3_)_3_, Sigma) and MPTMS (HS(CH_2_)_3_Si(OCH_3_)_3_, Sigma) for 1 h to provide specific functional groups. After the modification of functional groups, the samples were rinsed with ethanol several times and then heated in ethanol in an oven at 60 °C for 5 min. All the samples were blown dry with pure N_2_ gas. The APTMS and MPTMSSAMs modified NW-MOSFETs were ready for pH sensing by using electrical measurement and for constructing a biointerface by further immobilizing biomolecules. The biointerface was created by immersing APTMS or MPTMS modified NW-MOSFETs in 1-ethyl-3-(3-dimethylaminopropyl) carbodiimide (EDC) and N-hydroxysuccinimide (NHS) citrate buffered solution of antibody against prostate-specific antigen (anti-PSA, Ortho-Clinical Diagnostics Johnson & Johnson, 34 kDa). The EDC-NHS was utilized to only activate terminal carboxyl (-COOH) group of antibody [16,20] and maintain exposure of its active site for antigen binding.

### 2.3. Electrical Measurement for pH and Protein Sensings

Figure 1(b) shows the setup of the electrical measurement system used in our study. Electrical measurement was applied to record the signals from NW-MOSFETs for examining different pH solutions and protein-protein interactions in detecting PSA antigen. The variations of voltage signal of unmodified and modified NW-MOSFETs were independently measured. A bias voltage was applied to bulk silicon and source in relation to Ag/AgCl reference electrode (ALS, RE-1S) on ground potential in the solution. The negative bias voltage −10 V was applied. The voltage between drain and source was kept at −1 V, the source-drain current was −3 μA with a transconductance of about 300 nS. During the electrical measurement, the source-drain current was fed into a current-voltage converter. After amplification and filtering, the signal was digitized and read into a computer. Phosphate buffered saline (PBS) solutions at different pH values were individually introduced to unmodified, APTMS, or MPTMS modified NW-MOSFETs. In addition, PSA-antigen (VITROS PSA Calibrators, Ortho-Clinical Diagnostics Johnson & Johnson, Rochester, NY, USA) solutions of varied concentrations, human PSA in physiological buffer solution, contained antimicrobial agent and bovine serum albumin (BSA), were prepared for protein sensing. The acquired data were displayed in real-time.

### 2.4. AFM Scanning

AFM (Veeco Dimension 5000 Scanning Probe Microscope) was used to scan the surface morphology of the each-step modification of silicon surface to respond to the results of electrical measurement of NW-MOSFET. The AFM images were collected in tapping mode at a scanning frequency of 0.5 Hz. The Si tips (Nanosensors, PointProbePlus-RT-NCHR, tip curvature radius < 7 nm) of a resonance frequency of 200–500 kHz were utilized to scan the 3D morphology and roughness analysis of the surface.

## 3. Results and Discussion

### 3.1. Electrical Measurement

**Figure 2.**
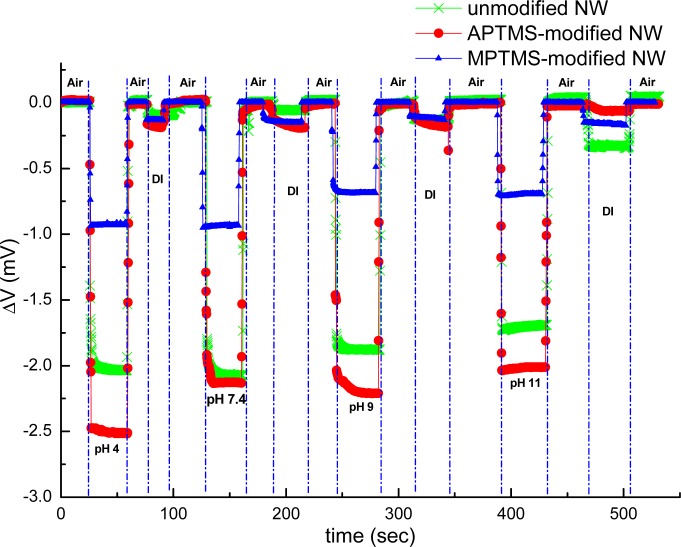
Electrical measurements for pH sensing were recorded by using unmodified (green line with crisscross symbols), APTMS-modified (red line with circle dots), and MPTMS-modified (blue line with triangle symbols) NW-MOSFETs.

The external localized field of the tested samples influenced carrier distribution in the near surface region of NW-MOSFETs. In our experiment setup, the recorded current between source and drain was fed into a current-voltage convertor; thereupon our final recorded signal was expressed in volts. Electrical measurement was used to verify that the varied functional groups pre- and postfunctionalization would have great impact on the sensing response and sensitivity of NW-MOSFETs in our study. The results of pH sensing for PBS solutions at different pH values using unmodified, APTMS and MPTMS modified NW-MOSFETs are shown in Figure 2.

**Figure 3.**
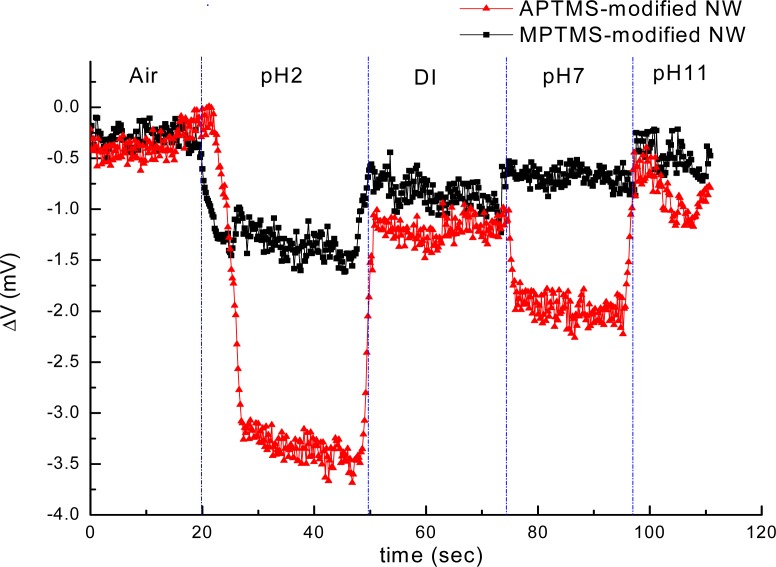
PH sensing was recorded by using APTMS (red line with triangle symbols) and MPTMS (black line with square symbols) modified NW-MOSFETs at pH values of 2, 7, and 11, respectively.

In contrast to MPTMS modified NW-MOSFETs, voltage changes of unmodified and APTMS modified NW-MOSFETs were significantly enhanced in various pH solutions. Voltage changes of pH 4 were found at 2.0 mV, 2.5 mV, and 0.9 mV on unmodified, APTMS-modified and MPTMS-modified NW-MOSFETs, respectively. In addition, above 2-fold signal changes on unmodified and APTMS-modified NW-MOSFETs were discovered in comparison with MPTMS modified NW-MOSFETs. It has been demonstrated that MPTMS-SAM modified on silicon NW-MOSFETs might form disulfide bonding between the mercapto groups of MPTMS [21]. As a consequence of the lower number of mercaptan groups of MPTMS on NWs, relatively minor signal responses to varied pH solutions are seen in Figure 2. On the other hand, our pH sensing results confirmed that the hydroxyl groups of the native oxide on silicon NW-MOSFETs and amino groups of APTMS-modified NW-MOSFETs can govern the protonation and deprotonation reactions in different pH solutions [4]. Since the hydroxyl groups and amino groups are sensitive to protonation and deprotonation [4,22], they respond to pH with a sizable signal change. In contrast to unmodified NWs (green line with crisscross symbols in Figure 2), the signal of APTMS-SAM modified NW-MOSFETs in Figure 2 (red line with circle dots) was enhanced slightly and shown APTMS-SAM did not deteriorate the sensing signal of NW-MOSFETs. It is worth mentioning that the voltage would return back to the initial level after each step of measurement on either unmodified or modified NW-MOSFETs. In Figure 3, we find the amino groups of APTMS promisingly improved the sensitivity of NW-MOSFET at pH values of 2, 7, and 11 in contrast with MPTMS. Furthermore, pH 2 solution protonated the surface of APTMS-modified NW-MOSFETs and resulted in significantly increasing voltage change in p-type devices [4]. Relatively smaller signal of MPTMS-modified NWs may be attributed to less well-ordered functional mercapto groups [21] and rather hydrophobic property [23]. The experiment of pH sensing showed our MOSFETs well performed in normal PBS.

### 3.2. Effect of Varied Functional Groups on Protein Sensing

**Figure 4.**
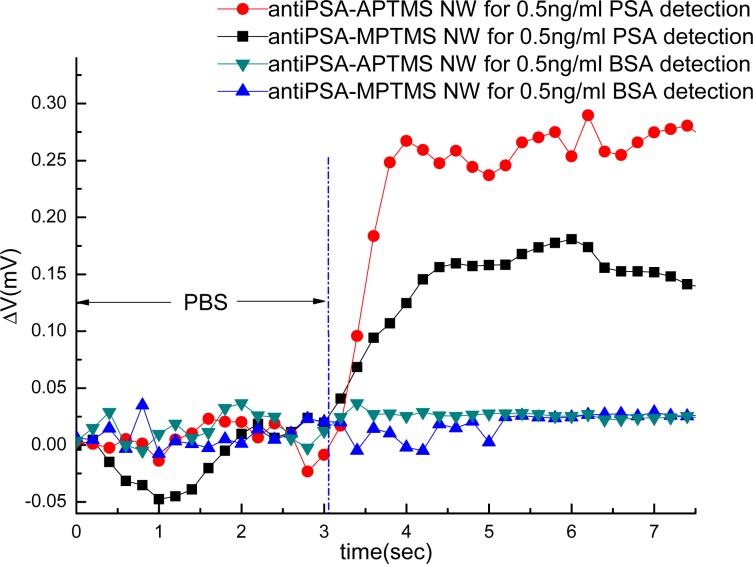
The detection of 0.5 ng/mL PSA and 0.5 ng/mL BSA using anti-PSA-APTMS and anti-PSA-MPTMS modified NW-MOSFETs.

In order to observe the effect of varied functional groups on protein sensing, anti-PSAs were independently immobilized on APTMS- or MPTMS-modified NW-MOSFETs. According to the general guide of PSA tests [18], the tested values found to be 4 ng/mL or less are regarded as normal. In our study, we utilized various concentrations of subthreshold-value PSA solutions, such as 0.25 ng/mL, 0.5 ng/mL and 1 ng/mL in physiological buffer solution, to demonstrate the sensitivity of our modified NW-MOSFETs. First, 0.5 ng/mL PSA was introduced to anti-PSA-APTMS and anti-PSA-MPTMS-modified NW-MOSFETs, the voltage changes were respectively increased to around 0.27 and 0.16 mV (Figure 4). Here, the interactions of PSA and biointerfaces, anti-PSA-APTMS and anti-PSA-MPTMS, were dynamically observed. For the protein-binding selectivity of anti-PSA-APTMS-and anti-PSA-MPTMS-modified NW-MOSFETs was tested by choosing 0.5 ng/mL BSA (pI∼4.6) as a negative control. In Figure 4, both anti-PSA-APTMS- and anti-PSA-MPTMS-modified NW-MOSFETs showed no significant voltage change to 0.5 ng/mL BSA (negatively charged at pH 7.4). In addition, the binding specificity of our anti-PSA-APTMS- and anti-PSA-MPTMS-modified NW-MOSFETs also manifested functionality in detecting specific biomedical molecules without compromising the concentration of buffer solution. Besides, we found the biointerface of anti-PSA-APTMS contributed better signal than anti-PSA-MPTMS. The immobilization of anti-PSA on APTMS-modified NWs rendered a more functional biointerface for better signal in PSA sensing.

**Figure 5.**
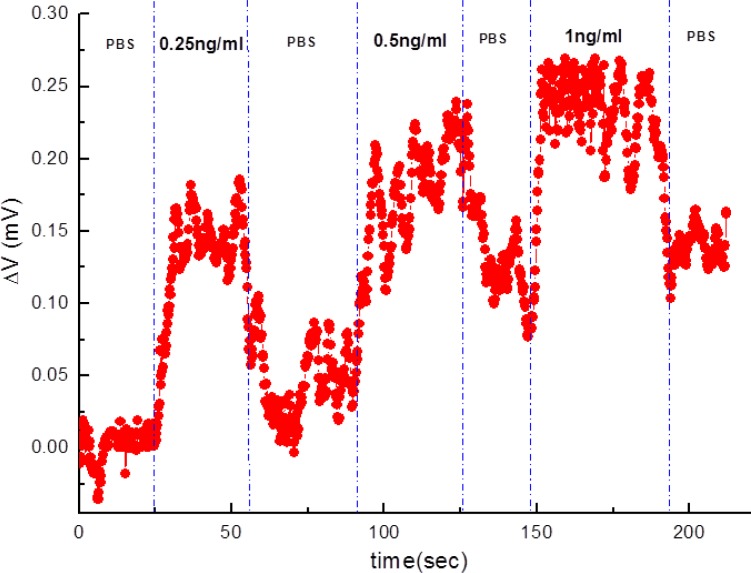
PSA sensing at different concentration by using the same anti-PSA-APTMS modified NW-MOSFET.

Furthermore, varied concentrations of subthreshold-value PSA solutions were detected on the same anti-PSA-APTMS-modified NW-MOSFET (Figure 5). Figure 5 shows that the anti-PSA-APTMS-modified NW-MOSFET were functional and sensitive to the lower concentration of 0.25 ng/mL PSA. A significant variation for PSA detection occurred at the concentrations of 0.25 ng/mL and 0.5 ng/mL on the same anti-PSA-APTMS-modified NW-MOSFET. Detection of 1 ng/mL PSA came after other two PSA solutions showed almost the same magnitude of response as 0.5 ng/mL PSA. Limited anti-PSAs on APTMS-modified NW-MOSFET corresponded to PSAs after PSA flowed through the surface of NW. Due to these limited antibodies on the surface of SAM-modified NWs, the detection signals finally reached saturation, even though higher concentrations of the corresponding antigens occurred on the anti-PSA-APTMS-modified NW-MOSFET. In addition, the levels of PBS after each PSA sensing were different in Figure 5. Since the interaction between antibody and antigen is very strong, it is difficult to disengage antigen from the corresponding antibody by using PBS [8]. Therefore, the level of PBS after each PSA detection was higher than the originals.

### 3.3. Verification of the Interaction of Biointerface and PSA

**Figure 6.**
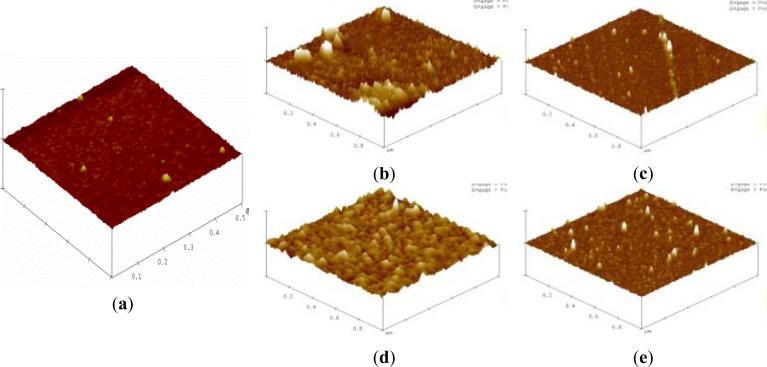
AFM images of (**a**) unmodified, (**b**) APTMS, (**c**) MPTMS, (**d**) anti-PSA-APTMS, and (**e**) anti-PSA-MPTMS modified silicon surfaces.

Silicon substrates were applied for verifying the overall interaction of biointerfaces and PSA on silicon NW-MOSFETs. Surface topographies of the silicon substrates before and after each step of the modifications were scanned by AFM and are shown in Figure 6. The AFM image of an unmodified silicon substrate prior to the surface modifications is displayed in Figure 6(a). After functionalization of APTMS and MPTMS on the silicon surfaces, rough topographies were observed in Figure 6(b) and (c), respectively. In comparison with MPTMS, APTMS could form an approximately uniform SAM on the silicon surface and provide functional amino (-NH_2_) groups [8] for later immobilizing relatively high amounts of biomolecules in Figure 6(d). In Figure 6(c), lower amount of MPTMS might be attributed to the formation of disulfide bonding between the mercapto groups of MPTMS [21]. Those disulfide bonds further influenced the organization of MPTMS-SAM on the surface. Since EDC-NHS citrate buffered solutions were used to activate the carboxyl groups of biomolecules [16,20], the illustrative AFM image of Figure 6(d) apparently shows anti-PSA was highly condensed on APTMS-SAM- modified silicon NW-MOSFETs. Taking advantage of EDC-NHS, anti-PSAs were certainly immobilized through covalent amide bonds and then exposed to the corresponding antigens. On the other hand, the immobilization of anti-PSA on MPTMS is totally different from APTMS since the MPTMS-modified surface would not undergo amidation with the EDC-NHS-activated antibodies. The possible immobilization of anti-PSA on MPTMS may be ascribed to the formation of disulfide bonds between restricted available mercapto groups and anti-PSAs. It is of interest to note that the amounts of anti-PSAs on MPTMS are less than those on APTMS in Figure 6(d) and (e). As a consequence of the lower anti-PSAs on MPTMS, smaller voltage changes were then observed in Figure 4 when the anti-PSA-MPTMS-modified NW-MOSFET sensed PSA. Overall, we found that anti-PSA with APTMS SAM showed better efficiency in the formation of biointerfaces on the silicon NW-MOSFETs. These relatively high amounts of anti-PSA on the APTMS-SAM-modified silicon NW-MOSFETs may result in better electrical signal performance when PSA contacted the corresponding anti-PSA (Figure 4).

**Figure 7.**
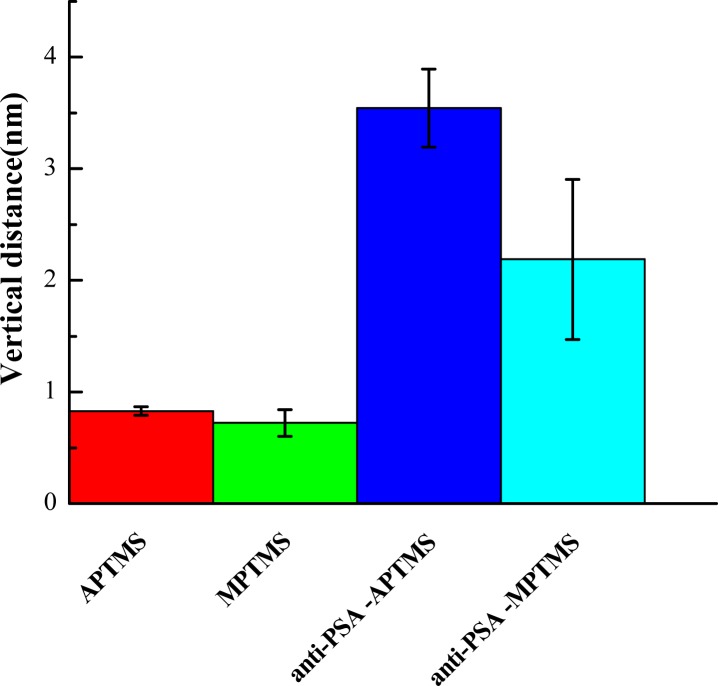
Thickness anlaysis for each-step modification on silicon by using AFM scanning technique. Error bars represent mean ± SEM (n = 6).

In AFM scanning, the changes of thickness in each modification step are shown in Figure 7. In order to measure the thickness of the SAMs, we analyzed the step profile of the APTMS and MPTMS modified silicon surfaces. The thicknesses of APTMS SAM and MPTMS SAM are about 0.83 nm and 0.72 nm in Figure 7.

Moreover, it is noted that the thickness of the functional biointerface constituted by anti-PSA on the APTMS-modified silicon surface increased to around 3.55 nm. In the parallel examination of anti-PSA on MPTMS, the thickness changed to around 2.19 nm. The reason for the varied thicknesses of anti-PSA on APTMS- and MPTMS-modified surfaces might be due to the different binding mechanisms which were discussed above. APTMS SAM on the SiNW-FET would reveal amino (-NH_2_) groups and further form a biointerface with amide (-CONH-) bonds after the carboxylic groups (-COOH) of other biomolecules were activated by EDC-NHS [16,20]. By contrast, mercapto (-SH) groups were exposed on MPTMS SAM-modified SiNW-FET and might find it difficult to interact with EDC-NHS activated biomolecules. Accordingly, the amounts of anti-PSA were relatively more on APTMS modified substrates. The successful and efficient immobilization of anti-PSA on APTMS-modified NWs rendered a functional biointerface for better signal in PSA sensing. It also verified that the adequate functional group of SAM modification on silicon NW-MOSFETs can improve the sensitivity of protein sensing.

## 4. Conclusions

Adequate modification could provide a biocompatible and functional interface. The idea of “biointerface” gave us an insight into future development of biosensors. Electrical measurement showed that modified NWs were still functionable and also sensitive to the external environmental changes. The result of pH sensing showed that amino groups of APTMS-modified NW-MOSFETs would easily protonate and deprotonate in different pH solutions in comparison with the lesser number of mercapto groups of MPTMS-modified NW-MOSFETs. Our created biointerface on silicon NW-MOSFET could successfully record protein interactions and possess specificity. Antigen-antibody reactions also verified that the adequate modification, such as APTMS-SAM, improved the overall sensitivity of silicon NW-MOSFETs. AFM examination demonstrated the modification can influence the electrical signals. Those AFM images showed better uniform arrangement of APTMS on silicon NW-MOSFETs and relatively higher amounts of antibodies on APTMS modified substrates. In conclusion, adequate modification could not only provide a biocompatible and functional interface, but also revolutionize medical applications of bioelectronics in the future.
